# Fatal pulmonary embolism following injectable gluteal filler usage: a case report

**DOI:** 10.1186/s43044-023-00415-9

**Published:** 2023-10-10

**Authors:** Sameh Shaheen, Ahmed Al-Habbaa, Mohamed Saeid Riad, Ahmed Saied Mandour, Mahmoud Ali Elzeny, Khaled Alnady

**Affiliations:** 1 Ain-Shams University, Faculty of medicine, Cairo, Egypt; 2 Armed Forces College of Medicine (AFCM), Cardiology department, Cairo, Egypt; 3 Al-Azhar University, Faculty of medicine, Cairo, Egypt; 4 Present Address: Kobri El-Kobba Military Hospital, Cairo, Egypt; 5 Mostafa Kamel Military Hospital, Alexandria, Egypt; 6 Military Medical Academy, Cairo, Egypt

**Keywords:** Pulmonary embolism, Gluteal filler, Injectable fluid silicone, Case report

## Abstract

**Background:**

Despite the fact that injectable filler usage in the gluteal region has not been recommended in formal medical institutions, illegal procedures are performed in many clinics and beauty centers across Egypt. This case report illustrates the illegal practice culminating in a fatal complication.

**Case presentation:**

A 26-year-old female with no relevant medical history presented to the ER with acute onset shortness of breath. The complaint started 16 h before, with a rapidly progressive course, shortly after undergoing a gluteal filler injection at a center in Cairo. At ER, the patient was severely distressed, yet fully conscious and oriented. She was shocked (BP 70/40 mmHg), tachycardic (130 BPM), and tachypneic (30/min) with normal temperature. She had congested pulsating neck veins with positive Kussmaul sign. Chest auscultation revealed normal vesicular breathing with equal air entry and no adventitious sounds. Her O2 saturation was 60% on room air that improved to 85% on O2 mask. ECG showed sinus tachycardia. Echocardiography showed dilated right side, D-shaped septum with systolic flattening, dilated IVC, mild tricuspid regurgitation and estimated RV systolic pressure 53 mmHg. Her ABG showed compensated metabolic acidosis with elevated lactate level. At the ICU, CVP was 18 mmHg. Saline infusion was continued along with noradrenaline infusion initiation. A provisional diagnosis of high-risk pulmonary embolism was made, though CT pulmonary angiography was not available. Accordingly, thrombolytic therapy was initiated with alteplase (100 mg) over 2 h. Also, a dose of pulse steroids (methylprednisolone 200 mg) was given. Chest X-ray showed bilateral heterogenous opacity and ABG showed deteriorating hypoxia and combined metabolic and respiratory acidosis. The patient was intubated upon deterioration of conscious level and was put on mechanical ventilation. Her ET tube showed frequent blood-tinged secretions. Echocardiography showed more right-side dilatation that was consistent with deterioration of clinical status. Three hours after admission the patient developed cardiac arrest and died 2 h later.

**Conclusions:**

This case report highlights the dangers associated with injectable filler usage in the gluteal region. Physicians and patients should be aware of the possible complications and how to avoid it.

## Background

Despite the fact that injectable filler usage in the gluteal region has not been recommended in formal medical institutions, illegal procedures are performed in many clinics and beauty centers across Egypt. This case report illustrates the illegal practice culminating in a fatal complication.

## Case presentation

A 26-year-old female with no relevant medical history and no special habits of medical importance presented to the ER with acute onset shortness of breath. The complaint started 16 h before, with a rapidly progressive course, shortly after undergoing a gluteal filler injection at a center in Cairo. The patient reported that she had 6 vials [3–5 ml each] and that it was planned for larger amount of injection but was interrupted as the patient developed symptoms.

**Fig. 1:**
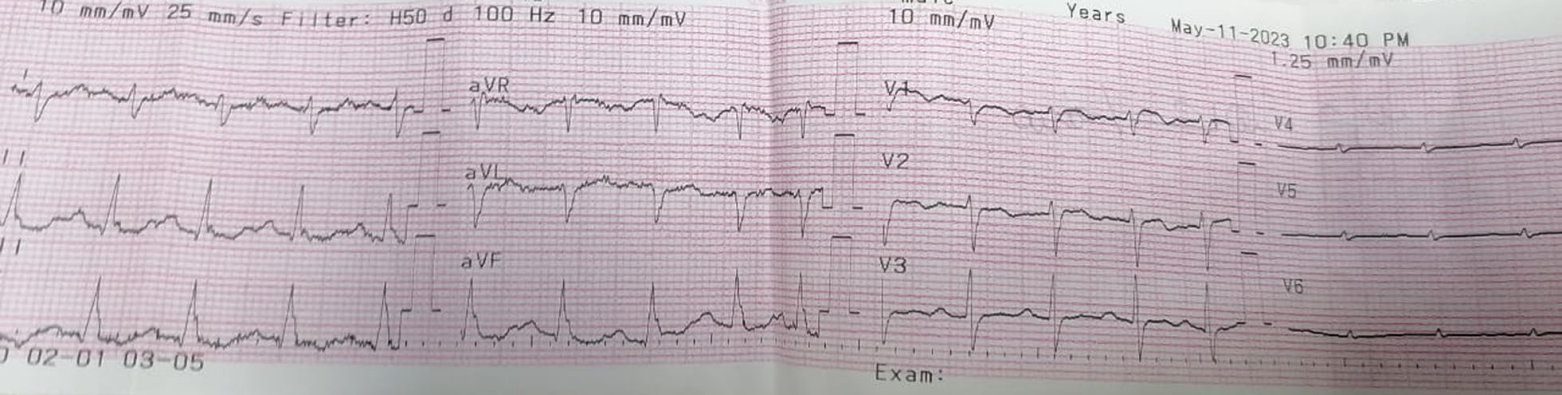
Patient ECG showing sinus tachycardia and right axis deviation

**Fig. 2:**
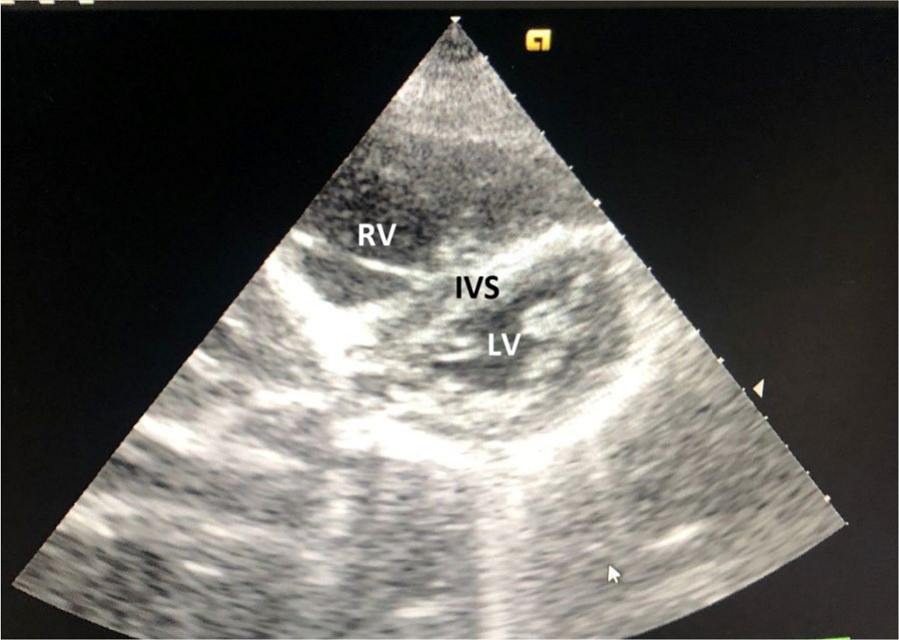
Patient echocardiogram at ER showing dilated right ventricle and D-shaped interventricular septum with systolic flattening

**Fig. 3:**
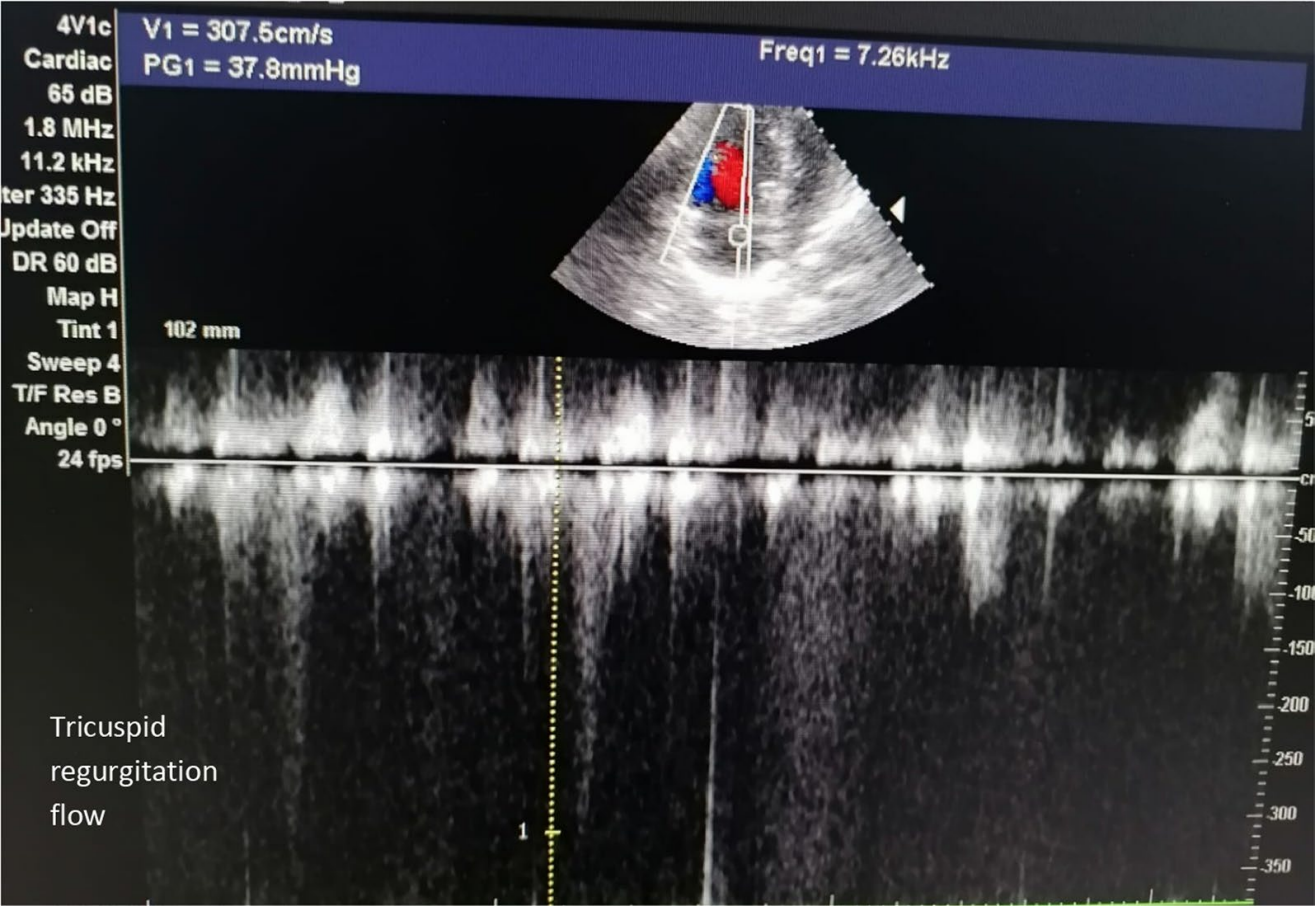
Patient echocardiogram at ER showing mild tricuspid regurgitation and estimated right ventricular systolic pressure of about 53 mmHg

At our ER, the patient was severely distressed, yet fully conscious and oriented. She was shocked (BP 70/40 mmHg), tachycardic (130 BPM), and tachypneic (30/min) with normal temperature. She had congested pulsating neck veins with positive Kussmaul sign. Her peripheral arterial pulses were faint and her capillary refill time was delayed. Chest auscultation revealed normal vesicular breathing with equal air entry and no adventitious sounds. Her O2 saturation was 60% on room air that improved to 85% on O2 mask. Her ECG showed sinus tachycardia (Fig. 1). Her echocardiography at ER showed dilated right ventricle and D-shaped interventricular septum with systolic flattening (Fig. 2), dilated IVC, mild tricuspid regurgitation and estimated right ventricular systolic pressure of about 53 mmHg (Fig. 3). Her ABG showed compensated metabolic acidosis with elevated lactate level. At ER, she received IV saline bolus of 500 ml with 5000 IU of UFH and was urgently transferred to the ICU.

**Fig. 4:**
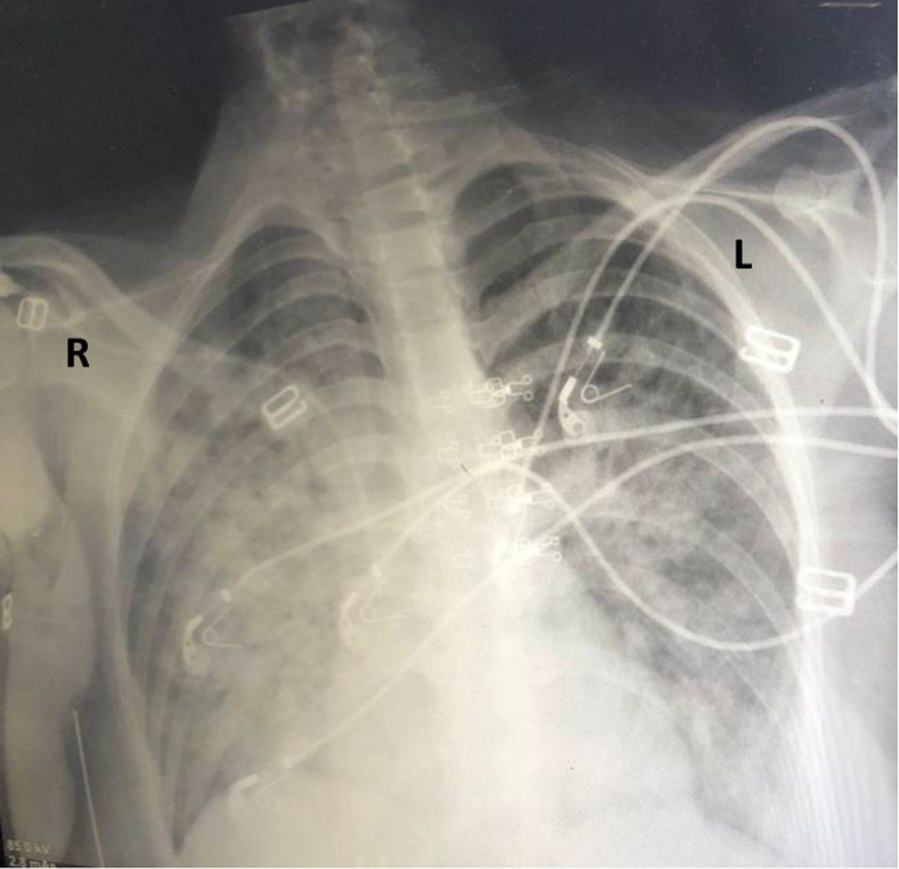
Patient chest X-ray in the ICU showing bilateral heterogenous infiltration and picture of ARDS

At the ICU, central venous line was inserted. CVP was 18 mmHg. Saline infusion was continued along with noradrenaline infusion initiation. A provisional diagnosis of high-risk pulmonary embolism was made, though CT pulmonary angiography was not available. Accordingly, thrombolytic therapy was initiated with alteplase (100 mg) over 2 h. Also a dose of pulse steroids (methylprednisolone 200 mg) was given. The patient deteriorated meanwhile. Her chest X-ray showed bilateral heterogenous infiltration and picture of ARDS (Fig. 4) and her serial ABG showed deteriorating hypoxia and combined metabolic and respiratory acidosis. The patient was intubated upon deterioration of conscious level and was put on mechanical ventilation (pressure support). Her ET tube afterward showed frequent blood-tinged secretions. Echocardiography after thrombolysis showed more right-side dilatation that was consistent with deterioration of clinical status. Three hours after admission the patient developed cardiac arrest on pulseless electrical activity. Advanced life support was done and ROSC after 30 min. Vasopressor dose was elevated, adrenaline infusion was added and the patient was put on therapeutic hypothermia. The patient developed cardiac arrest 3 times and died 2 h after the first arrest.

## Time line

**Table:** 

16 h before admission	Gluteal filling procedure and immediate start of complaints
Admission	Severe shortness of breath and cardiogenic shock
3 h after admission	Patient developed cardiac arrest
2 h later	Patient died

## Discussion

According to the American Society for Aesthetic Plastic Surgery (ASAPS), the placement of dermal fillers is the second most commonly performed minimally invasive cosmetic procedure in the USA. ASAPS data show that there is an increase in buttock augmentation procedures from 2015 to 2019 by 90.3% [1].

Gluteal cosmetic procedures can be surgical silicone implants, autologous fat grafting or local injection of injectable fillers. The most commonly used injectable for buttock augmentation includes: Hyaluronic acid, fluid silicone, Polyalkylimide Gel, Polyacrylamide Gel (PAAG) and Polymethylmethacrylate (PMMA) [2].

Injectable silicone is not approved by the US Food and Drug Administration for any esthetic procedure including facial and body contouring or enhancement [3].

Complications of gluteal cosmetic procedures include gluteal abscess, skin ulceration, granuloma, foreign body migration, acute and late onset pneumonitis and pulmonary embolism. Though rare, embolization of soft dermal fillers resulting in pulmonary embolism and death has been reported [4].

According to the ASERF task force, despite the growing popularity of gluteal fat grafting, significantly higher mortality rates appear to be associated with gluteal fat grafting than with any other esthetic surgical procedure. The Multi-Society Task Force for Safety in Gluteal Fat Grafting (ASAPS, ASPS, ISAPS, IFATS, ISPRES) recently released an urgent practice advisory in response to the alarming number of deaths still occurring from this procedure. The unusually high mortality rate from this cosmetic procedure is estimated to be as high as 1:3000, greater than any other cosmetic surgery [5].

Rapkiewicz et al. [6] presented a series of 10 deaths complicating gluteal procedures with the majority occurring in the setting of liposuction and gluteal fat transfer. They reported post-mortem autopsy findings of macroscopic pulmonary fat embolism.

Bejarano et al. [7] reported pulmonary complications related to the illegal use of injected silicone for cosmetic procedures. Silicone injection can cause complications similar to that seen in fat embolism inducing inflammation and immune response activation with consequent alveolar hemorrhage, diffuse alveolar damage and acute respiratory distress syndrome (ARDS).

Carolyn et al. [8] reported a case of acute pneumonitis after silicone injection for gluteal augmentation. The patient presented with hemoptysis, shortness of breath, and acute respiratory failure two days after the silicone injections. Her chest computed tomography (CT) showed predominantly basilar and peripheral Ground Glass Opacity (GGO) and bilateral pulmonary nodules. Her condition required ECMO and she improved with intravenous methylprednisolone 125 mg every 6 h.

Inayat et al. [9] reported that filler-induced non-thrombotic pulmonary embolism (NTPE) can be either mechanical obstruction of the pulmonary circulation or chemical inflammatory reaction with pneumonitis and alveolar hemorrhage. The precipitating factors include high-pressure injection, large filler volume injection, massage or trauma at the injection site and direct injection into a vein [10, 11].

Ng et al. [12] reported that, for decades, liquid injectable silicone has been used for correction of contour defect or soft-tissue augmentation. Medical-grade liquid silicone (polydimethylsiloxane) became the preferred inert material for cosmetic purpose due to its durability, lack of immunogenicity and thermal stability. However, it was found later that silicone can induce pulmonary embolism (silicone embolism syndrome: SES). It has been reported by several studies as a cause of acute pneumonitis with alveolar hemorrhage and ARDS [13].

Cases of NTPE occurs typically in young females with recent history of gluteal cosmetic procedures. Presentation is mostly within few hours after the procedure, but may be late, with no evidence of DVT. Symptoms will range from mild dyspnea, cough and expectoration up to extreme difficulty in breathing with hemoptysis and signs of acute respiratory failure. Hemodynamics can be stable or the patient might be shocked with tachypnea and tachycardia. Chest auscultation might reveal crepitations of inflammatory secretions. Blood gases will indicate hypoxia and mild hypercapnia. Chest X-ray will show extensive pneumonic infiltrates. A 2-D echocardiogram might reveal severe pulmonary hypertension and RV dysfunction. Lung computed tomography (CT) can show diffuse alveolar infiltrates, bilateral GGO and pleural effusion. CT pulmonary angiography might not show evidence of acute venous thromboembolism. Bronchoscopy will show diffuse alveolar hemorrhages with no overt source of bleeding. Bronchoalveolar lavage fluid cytology may show macrophages and mixed inflammatory cells and negative cultures. Trans-bronchial lung biopsy shows lung parenchyma with intra-alveolar hemorrhage, macrophages and non-refractile vacuole like structures [14].

Fat embolism syndrome (FES) that might occur with liposuction and autologous fat grafting is classically characterized by the combination of a triad: acute respiratory failure, neurologic abnormalities, and a petechial rash. Patients presenting to the ER with sudden alteration in mental status should be questioned for recent surgical or invasive cosmetic procedures. FES should be considered even if the patient has no petechial rash. Brain magnetic resonance images (MRI) and lung CT should be ordered for these patients. Patient might have multiple cerebral white lesions on brain MRI [15].

Compared to the amount of filler used in facial procedures [2–5 cc], commonly used fillers or autologous fat transfers in the gluteal region usually involve up to 600 cc in each buttock. This large amount of filler in the gluteus muscles increases the likelihood of embolization. Moreover, application of these fillers in vascular regions of the gluteus muscles make embolization more likely [16].

According to the Multi-Society Task Force for Safety in Gluteal Fat Grafting, pulmonary fat embolism is the most common cause of mortality. The mechanism is fat entering the venous circulation after penetration of the gluteal veins. Autopsy findings in all mortality cases universally showed fat within the gluteal muscle. The Task Force has therefore concluded that fat should never be placed in the muscle. Fat should only be placed in the subcutaneous tissue. Operators should avoid deep injection, should use wide-bore syringe and blunt cannula, aspirate prior to each injection and use ultrasound-guided injection. Patients interested in gluteal augmentation through fat grafting or filler procedures should be informed of the risk of pulmonary fat or filler embolism which might end in death. They should be informed about the alternatives of these procedures that include silicone implant-based gluteal augmentation [5].

Treatment of a complicated case of filler pulmonary embolization and pneumonitis is based on limited experience as there is no published consensus. Patients with severe respiratory symptoms might be admitted to ICU and treated with massive IV steroids and antibiotics. The role of anticoagulants or thrombolytic therapy is controversial. Sedation and prone position, non-invasive or even invasive mechanical ventilation might be needed. ECMO might be considered in patients complicated by hemodynamic shock or severe ARDS and acute respiratory failure.

## Conclusions

This case report highlights the eminent risk of illegal cosmetic procedures using liquid silicone for gluteal enlargement. Physicians should be aware of the possible complications. We need peer reviewed and well-controlled studies to assess the efficacy and safety of cosmetic gluteal filler injection. Until we have such studies, medical and legal authorities should prohibit these procedures and public advertisement should be prevented. Patients have the right to know the possible complications of these procedures.

## Data Availability

All data generated or analyzed during the management of this patient are included in this manuscript. The data and material are available in the archives of the hospital.

## Abbreviations

2-D: Two dimensional
ABG: Arterial blood gases
ARDS: Adult respiratory distress syndrome
ASAPS: American Society for Aesthetic Plastic Surgery
ASERF: Aesthetic Surgery Education and Research Foundation
ASPS: American Society for Plastic Surgery
BP: Blood pressure
BPM: Beats per minute
CT: Computed tomography
CVP: Central venous pressure
DVT: Deep venous thrombosis
ECG: Electrocardiogram
ECMO: Extracorporeal membrane oxygenation
ER: Emergency room
ET: Endotracheal
FES: Fat embolism syndrome
GGO: Ground glass opacities
ICU: Intensive care unit
IFATS: International Federation for Adipose Therapeutics and Science
ISAPS: International Society for Aesthetic Plastic Surgery
ISPRES: International Society of Plastic Regenerative Surgeons
IV: Intravenous
IVC: Inferior vena cava
IU: International unit
MRI: Magnetic resonance imaging
NTPE: Non-thrombotic pulmonary embolism
O2: Oxygen
PAAG: Polyacrylamide gel
PMMA: Polymethylmethacrylate
ROSC: Return of spontaneous circulation
SES: Silicone embolism syndrome
UFH: Unfractionated heparin
